# G-quadruplexes offer a conserved structural motif for NONO recruitment to NEAT1 architectural lncRNA

**DOI:** 10.1093/nar/gkaa475

**Published:** 2020-06-04

**Authors:** Eric A J Simko, Honghe Liu, Tao Zhang, Adan Velasquez, Shraddha Teli, Aaron R Haeusler, Jiou Wang

**Affiliations:** Department of Biochemistry and Molecular Biology, Johns Hopkins University Bloomberg School of Public Health, 615 N. Wolfe St, Baltimore, MD 21205, USA; Department of Neuroscience, Johns Hopkins University School of Medicine, Baltimore, MD 21205, USA; Department of Biochemistry and Molecular Biology, Johns Hopkins University Bloomberg School of Public Health, 615 N. Wolfe St, Baltimore, MD 21205, USA; Department of Neuroscience, Johns Hopkins University School of Medicine, Baltimore, MD 21205, USA; Department of Biochemistry and Molecular Biology, Johns Hopkins University Bloomberg School of Public Health, 615 N. Wolfe St, Baltimore, MD 21205, USA; Department of Neuroscience, Johns Hopkins University School of Medicine, Baltimore, MD 21205, USA; Department of Biochemistry and Molecular Biology, Johns Hopkins University Bloomberg School of Public Health, 615 N. Wolfe St, Baltimore, MD 21205, USA; Department of Neuroscience, Johns Hopkins University School of Medicine, Baltimore, MD 21205, USA; Department of Biochemistry and Molecular Biology, Johns Hopkins University Bloomberg School of Public Health, 615 N. Wolfe St, Baltimore, MD 21205, USA; Department of Neuroscience, Johns Hopkins University School of Medicine, Baltimore, MD 21205, USA; Department of Biochemistry and Molecular Biology, Johns Hopkins University Bloomberg School of Public Health, 615 N. Wolfe St, Baltimore, MD 21205, USA; Department of Neuroscience, Johns Hopkins University School of Medicine, Baltimore, MD 21205, USA; Department of Biochemistry and Molecular Biology, Johns Hopkins University Bloomberg School of Public Health, 615 N. Wolfe St, Baltimore, MD 21205, USA; Department of Neuroscience, Johns Hopkins University School of Medicine, Baltimore, MD 21205, USA

## Abstract

The long non-coding RNA NEAT1 serves as a scaffold for the assembly of paraspeckles, membraneless nuclear organelles involved in gene regulation. Paraspeckle assembly requires NEAT1 recruitment of the RNA-binding protein NONO, however the NEAT1 elements responsible for recruitment are unknown. Herein we present evidence that previously unrecognized structural features of NEAT1 serve an important role in these interactions. Led by the initial observation that NONO preferentially binds the G-quadruplex conformation of G-rich *C9orf72* repeat RNA, we find that G-quadruplex motifs are abundant and conserved features of NEAT1. Furthermore, we determine that NONO binds NEAT1 G-quadruplexes with structural specificity and provide evidence that G-quadruplex motifs mediate NONO-NEAT1 association, with NONO binding sites on NEAT1 corresponding largely to G-quadruplex motifs, and treatment with a G-quadruplex-disrupting small molecule causing dissociation of native NONO-NEAT1 complexes. Together, these findings position G-quadruplexes as a primary candidate for the NONO-recruiting elements of NEAT1 and provide a framework for further investigation into the role of G-quadruplexes in paraspeckle formation and function.

## INTRODUCTION

Membraneless organelles, commonly referred to as ‘granules’ or ‘bodies’, are distinct subcellular collections of macromolecules which are compartmentalized in the absence of an encapsulating lipid membrane (1). A variety of biological processes are executed by membraneless bodies. Production of certain ribosomal subunits, for instance, is carried out by the nucleolus, a subnuclear compartment containing ribosome biogenesis factors and encompassing ribosomal RNA gene clusters (2). Degradation of messenger RNA (mRNA) is regulated, in part, by dynamic cytoplasmic granules known as processing bodies (P-bodies) which consist primarily of mRNA decay factors and translationally repressed mRNAs (3). Emerging evidence that disruption of membraneless organelles is a common feature of age-related disease has made them a topic of widespread interest (4). In the absence of an encapsulating lipid membrane, formation and persistence of these bodies is based solely on interactions between and among their protein and nucleic acid components. The biochemical principles guiding these interactions are therefore a topic of particular importance (5). RNA components have emerged as important factors, scaffolding the assembly of many cytoplasmic and nuclear bodies (5,6). In some cases, RNA components consist of general classes of RNA such as spliced mRNAs, which seed formation of splicing speckles (7). Many nuclear bodies require specific species of long non-coding RNA (lncRNA) such as the satellite III (satIII) repeat transcripts which seed formation of nuclear stress bodies (7). SatIII transcripts and other lncRNAs with critical roles in the formation and function of nuclear bodies have been termed architectural RNAs (arcRNAs) (8).

Paraspeckles, nuclear bodies built on the arcRNA NEAT1, regulate diverse physiological processes, with roles ranging from those in the nervous system (9–11) to those in female fertility (12–14). At the cellular level, a variety of stresses induce paraspeckle sequestration or release of specific components to regulate gene expression at the levels of transcription (12,15,16), nuclear export (9,17–20), and microRNA biogenesis (21). With a multi-step assembly process (22–29) producing a complex and well-defined structural arrangement (24,30), paraspeckles provide an ideal model for understanding the processes behind construction and persistence of membraneless bodes built on RNA scaffolding.

Two mature isoforms are produced from the NEAT1 gene: NEAT1_1, ∼3,700 nucleotides in length, and NEAT1_2, extending beyond the NEAT1_1 termination site for a total length of ∼22,700 nucleotides (31,32). NEAT1_2 is an essential component of paraspeckles, while the short isoform is dispensable (26); ‘NEAT1’ herein refers to the long isoform unless otherwise specified. NEAT1 ribonucleoprotein particles (RNPs), the primary subunits of paraspeckles, are produced by the co-transcriptional assembly of a subset of paraspeckle proteins onto individual NEAT1 transcripts. NEAT1 RNP subunits are then assembled with additional protein components to produce mature paraspeckles (24–28), with each paraspeckle constructed from, on average, ∼50 primary NEAT1 RNPs (29).

Non-POU domain-containing octamer-binding protein (NONO) and splicing factor proline- and glutamine-rich (SFPQ), both members of the *Drosophila* behaviour human splicing (DBHS) protein family, play a critical role in paraspeckle formation. The first step of primary RNP formation is recruitment of NONO and SFPQ to nascent NEAT1 transcripts, both stabilizing NEAT1 and providing the foundation necessary for recruitment of the additional protein components which facilitate subsequent steps in assembly and maturation (6,24,25,33). While NONO and SFPQ have been shown to interact directly with NEAT1 (18,23,25,31), the specific RNA elements responsible for their recruitment remain unknown (34–36). Identification of these functional recruitment elements has been complicated by low conservation of the NEAT1 sequence (32,37). In lieu of well-defined primary sequence motifs, it has been suggested that these critical interactions could be mediated by secondary structures which have yet to be recognized (34,36,38).

Formed by nucleic acids containing at least four proximal runs of two or more guanines, G-quadruplexes are four-stranded secondary structures stabilized by non-Watson-Crick base pairing. Two or more planar G-tetrads—guanine quartets formed by Hoogsteen base pairing—are stabilized by stacking one atop another, often with added stabilization from monovalent cations such as potassium (39,40). Due to steric constraints (41), G-quadruplexes formed by RNA typically assume a ‘parallel’ topology where all four guanine runs are oriented with uniform 5′ to 3′ directionality (42). Functioning largely through specific recognition by RNA-binding proteins, RNA G-quadruplexes regulate biological processes such as pre-mRNA processing (43–47), microRNA biogenesis (48,49), and translation (50–56). In addition to regulating basic cellular processes, G-quadruplex-forming RNAs have been linked to diseases ranging from cancer to neurodegeneration (57). A mutation in the *C9orf72* gene linked to neurodegenerative phenotypes results in the toxic accumulation of an RNA species consisting of hundreds of tandem repeats of the hexanucleotide sequence ‘GGGGCC’ (58–61) which we and others have shown forms stable G-quadruplexes (62–64). Toxic effects of GGGGCC repeat RNA accumulation are due in part to aberrant binding and sequestration of cellular proteins (65), positioning the focus of significant efforts on identification of proteins which interact with this RNA.

In previous work, we identified protein interactors of GGGGCC repeat RNA using a proteomic approach designed specifically to reveal proteins binding the RNA with structural discrimination (62). Capitalizing on an instance of structure-specific binding revealed by our prior assay, herein we uncover widespread G-quadruplexes along the length of NEAT1, demonstrate the importance of these structures to interactions critical for paraspeckle biogenesis, and find that enrichment in G-quadruplexes is a conserved feature of NEAT1. In line with earlier predictions, we put forward this conserved structural feature as the first proposed candidate to begin answering the question of which specific NEAT1 elements recruit paraspeckle proteins to seed the initial stages of paraspeckle assembly.

## MATERIALS AND METHODS

### Oligonucleotide synthesis and annealing

All oligonucleotides used in this study were purchased from Integrated DNA Technologies, Inc. RNA was synthesized at 100 nmol scale, purified by RNase-free HPLC, and confirmed by electrospray ionization mass spectrometry. Fluorescently-labelled RNA contained a Cy5 molecule on the 3′ end. Oligonucleotides used in the RNA pulldown experiment were biotinylated on the 3′ end.

At concentrations given for individual experiments, RNA was pre-annealed in 10 mM Tris-HCl pH 7.5, with or without 100 mM KCl. Annealing consisted of heating at 95°C to melt followed by cooling over 90 min and was performed in an S1000 thermal cycler (BioRad) programmed as follows: (i) 95°C for 5 min; (ii) 95°C for 1 min 9 s with -1.0°C decrement/cycle; (iii) go to step (ii) 70 times; (iv) hold at 4°C. Annealing for full-length NONO EMSAs used a similar protocol, except cooled from 95°C to 4°C over 12 h with 910 cycles of -0.1°C decrement/cycle and 41 s/cycle.

### RNA pulldown

RNA pulldown was performed as previously described (62) with minor modifications. For each pulldown, 1.2 nmol biotinylated RNA oligonucleotide was annealed at 6 μM in the manner described above. For each pulldown, PureProteome Streptavidin Magnetic Beads (EMD Millipore) from 200 μl slurry were washed twice in 1 ml RNA Pulldown (RP) buffer (10 mM HEPES pH 7.0, 200 mM KCl, 1% Triton X-100, 1 mM MgCl_2_, 1 mM DTT, 1× cOmplete, EDTA-free Protease Inhibitor Cocktail (Roche)). All bead incubations were carried out at 4°C in a 115 VAC 18 rpm Tube Rotator (Cole-Parmer), and all washes were incubated for 5 min. Beads were resuspended in 0.8 ml RP buffer, 1.2 nmol annealed biotinylated RNA oligonucleotides were added to each, and conjugation was carried out at 4°C for 50 min before washing twice in 1 ml RP buffer.

Stock lysate for each replicate was produced from one 10 cm dish of HEK293T cells harvested at ∼90% confluency. Cells were detached using TrypLE Express (Gibco), centrifuged at 150 g for 5 min to pellet, and washed twice with 10 ml ice-cold PBS prior to lysis. Pellets were resuspended in 1 ml RP buffer. To lyse, resuspended cells were drawn into and forcefully expelled from 1 ml micropipette tips ten times and then incubated on ice for 20 min. Sonication was performed using a Bioruptor (Diagenode) with 15 ml tube adaptors. Two rounds of sonication were performed in ice-cold water, each round consisting of 5 min of 30 s/30 s on/off cycles on medium power. Lysate was centrifuged at 16,100 g for 10 min at 4°C and the soluble fraction was retained. Protein content of stock lysate was measured by bicinchoninic acid (BCA) assay (Pierce) following the manufacturer's protocol and lysate was diluted to 1 mg/ml in RP buffer for preclearing. 10 μg egg white avidin and 0.5 mg yeast tRNA were added for each milligram of protein and lysate was incubated rotating at 4°C for 1 h. After centrifuging at 16,100 g for 20 min at 4°C, the soluble fraction was retained and RNaseOUT Recombinant Ribonuclease Inhibitor (Invitrogen) was added to 200 units/ml.

Precleared lysate was diluted to 0.1 mg/ml protein equivalent in RP buffer. For each pulldown, RNA-conjugated beads were resuspended in 1 ml of 0.1 mg/ml precleared lysate and incubated for 1.5 h. Beads were washed twice with 1 ml RP buffer followed by a series of 250 μl washes with increasing salt (RP buffer supplemented to contain 0.4, 0.8 and 1.6 M KCl). To reduce non-specific binding and residual salt before eluting, beads were resuspended in 0.2 ml RP buffer and transferred to a clean 1.5 ml tube containing 0.8 ml RP buffer. This final wash was removed before resuspending beads in 250 μl of 1× Laemmli Sample Buffer (62.5 mM Tris-HCl pH 6.8, 10% glycerol, 2% SDS, 5% 2-mercaptoethanol, 0.002% bromophenol blue) and heating to 95°C for 10 min to elute.

The following modifications were made for mass spectrometry experiments: Each pulldown was scaled up by a factor of two and eluted in 100 μl of 1× Laemmli Sample Buffer. For preclearing, lysate was diluted to 2 mg/ml in RP buffer. Instead of egg white avidin, PureProteome Streptavidin Magnetic Beads (EMD Millipore) were used for preclearing at 350 μl slurry/ml of lysate.

### Tandem mass tag labelling and mass spectrometry

Protein extracts were alkylated with 250 mM iodoacetamide, diluted with 300 μl 8 M urea, and buffer exchanged over an Amicon Ultra 30 kDa MWCO spin cartridge (0.5 ml, Millipore) to remove SDS. Proteins on the filter were washed 3 times with 8 M urea followed by 3 times with 10 mM triethylammonium bicarbonate (TEAB) pH 7.5 and resuspended in 2 ml TEAB. Trypsin (400 μl, Promega, V5111) was added directly to the upper filter chamber before digesting overnight at 37°C. Following digestion, peptides were collected and dried by vacuum centrifugation.

For peptide labeling with isobaric mass tags, tandem mass tag (TMT) label reagents (0.8 mg vials, Thermo Scientific) were brought to room temperature before resuspension in 41 μl of anhydrous acetonitrile. Peptides were reconstituted with 100 μl of 100 mM TEAB. A randomized TMT reagent solution was added to each peptide sample and reactions were carried out at room temperature for 1 h before quenching with 8 μl of 5% hydroxlamine. Samples were combined, split into 10% and 90% aliquots, dried by vacuum centrifugation, and stored at -80°C.

Combined samples of TMT-labeled peptides were resuspended in 200 μl of 0.1% trifluoroacetic acid and desalted on an Oasis C18 plate (Waters). Desalted peptides were eluted with 10 mM TEAB in steps at 5, 10, 25 and 75% acetonitrile and dried.

Peptide fractions were analyzed by liquid chromatography interfaced with tandem mass spectrometry (LC-MS/MS) using an Easy-LC 1200 HPLC system (Thermo Scientific) interfaced with an Orbitrap Fusion Lumos Tribrid Mass Spectrometer (Thermo Scientific). Fractions were resuspended in 20 μl loading buffer (2% acetonitrile in 0.1% formic acid) and analyzed by reverse phase liquid chromatography coupled to tandem mass spectrometry. 20% of each fraction was loaded onto a C18 trap (S-10 μM, 120 Å, 75 μm × 2 cm, YMC) and subsequently separated on an in-house packed PicoFrit column (75 μm × 200 mm, 15 ± 1 μm tip, New Objective) with C18 phase (ReproSil-Pur C18-AQ, 3 μm, 120 Å, Dr Maisch GmbH) using a 2-90% acetonitrile gradient at 300 nl/min over 120 min. Eluting peptides were sprayed at 2.0 kV directly into the Lumos.

Survey scans (full MS) were acquired from 370 to 1700 *m*/*z* with data dependent monitoring with a 3 s cycle time. Each precursor was individually isolated in a 0.7 Da window and fragmented using HCD activation collision energy 39 and 15 s dynamic exclusion, first mass being 115 *m*/*z*. Precursor and fragment ions were analyzed at resolutions 120,000 and 30,000, respectively, with automatic gain control (AGC) target values at 4e5 with 50 ms maximum injection time (IT) and 1e5 with 120 ms maximum IT, respectively.

Isotopically resolved masses in precursor (MS) and fragmentation (MS/MS) spectra were processed in Proteome Discoverer software (v2.3, Thermo Scientific). All data were searched using Mascot (2.6.2, Matrix Science) against the 2017_Refseq 83 Human database. The following criteria were set for the database search: all species; trypsin as the enzyme, allowing one missed cleavage; cysteine carbamidomethylation and N-terminal TMT label as fixed modifications; TMT label on lysine, methionine oxidation, asparagine and glutamine deamidation as variable modifications. Mass tolerance was 5 ppm and 0.03 Da for Precursors and Peptides, respectively. Identifications from Mascot searches were filtered at 1% false-discovery rate confidence threshold based on a concatenated decoy database search using Proteome Discoverer. ‘Enrichment over beads’ is the mean of abundance ratios for the peptides of each protein, excluding peptides with shared sequences, with abundance ratio representing abundance of each peptide in the RNA pulldown over abundance in pulldown with beads. Known paraspeckle proteins (36) which were identified in the RNA pulldown were selected to generate a heat map using Proteome Discoverer. Heat map is based on abundance data, which was scaled before clustering (distance function: Euclidean; linkage method: complete).

### Protein expression and purification

NONO(53-312) was expressed and purified using a previously published protocol with modifications (66). 50 ml overnight starter cultures of Rosetta 2(DE3)pLysS (Novagen) transformed with pET-Duet-NONO_53-312 were used to seed 1 l cultures, which were grown at 37°C in lysogeny broth containing ampicillin (100 μg ml^-1^) and chloramphenicol (34 μg ml^-1^). At OD_600_ of 0.8-1.0, cultures were placed at 4°C for 1 h, then induced with 0.5 mM isopropyl β-d-1-thiogalactopyranoside and grown overnight at 16°C. Bacterial pellets were harvested by centrifugation at 17,000 g for 20 minutes at 4°C and stored at -80°C until purification.

Pellets were thawed at 4°C and resuspended at 3.5 ml/g of pellet in Lysis Buffer (50 mM Tris-HCl pH 7.5, 250 mM NaCl, 25 mM imidazole, 10% glycerol) supplemented with cOmplete, EDTA-free Protease Inhibitor Cocktail (Roche) and with Pierce Universal Nuclease for Cell Lysis (Thermo Scientific) at 1:10,000. Resuspended bacteria were lysed by two passes through a French press at 1000 psi and clarified by centrifugation at 45,000 g for 30 min at 4°C. Clarified lysate was passed through an 0.45 μm filter prior to purification.

Initial purification by immobilized metal affinity chromatography (IMAC) used a 5 ml HisTrap (GE Healthcare). Lysate was applied to a column pre-equilibrated in 5 column volumes (CV) of Lysis Buffer at 1 ml/min. The column was washed with Lysis Buffer until absorbance returned to baseline and protein was eluted with a gradient of 25-500 mM imidazole in Lysis Buffer over 10 CV. IMAC fractions containing NONO(53–312) were pooled and then diluted 1:3 in SEC Buffer (20 mM Tris-HCl pH 7.5, 250 mM KCl, 0.5 mM EDTA, 50 mM l-proline). To remove the histidine affinity tag, tobacco etch virus (TEV) protease (Gene and Cell Technologies) was added by weight at 1:10 TEV:target and reactions were supplemented with 1 mM DTT. TEV digestion was carried out overnight at 25°C. Sample was centrifuged at 70,000 g for 15 min at 4°C to remove precipitate and supernatant was passed through 0.45 μm filter. Buffer exchange into RvsIMAC Binding Buffer (20 mM Tris-HCl pH 7.5, 250 mM KCl, 50 mM l-proline) was performed using a HiPrep Desalt 26/10 column (GE Healthcare). Reverse IMAC was performed on pooled fractions containing NONO(53-312) using a 5 ml HisTrap (GE Healthcare) equilibrated in RvsIMAC Buffer and flow-through fractions containing NONO(53-312) were pooled. Protein was concentrated using a Centrifugal Filter Unit with a molecular weight cut-off of 10K (Millipore). Concentrated sample was centrifuged at 20,000 g for 10 min at 4°C and soluble fraction was saved. Size exclusion chromatography was performed using a Superdex 200 10/300 GL column (GE Healthcare) pre-equilibrated in SEC Buffer. 400 μl sample was injected using a 1 ml sample loop and chromatography was run using SEC Buffer with a flow rate of 0.4 ml/min. Fractions containing NONO(53-312) were pooled and centrifuged at 20,000 g for 15 min at 4°C and supernatant was collected. Protein concentration in supernatant was determined from the absorption at 280 nm, measured using a Nanodrop 2000 (Thermo Scientific), using an estimated molar extinction coefficient of 11,585 M^-1^cm^-1^. Protein was diluted to 25 μM in SEC Buffer, aliquoted, and saved at -80°C.

Full-length NONO included a MYC/DDK-tag, was commercially produced and purified from HEK293T cells (Origene, TP306688), and was diluted for titrations in storage buffer (25 mM Tris-HCl pH 7.3, 100 mM glycine, 10% glycerol).

### Electrophoretic mobility shift assay

Cy5-labeled RNA oligonucleotides were annealed at 100 nM in the presence or absence of potassium. Binding reactions included 10 nM labelled RNA and recombinant protein at given concentrations. For NONO(53-312), binding reactions had a final buffer composition of 20 mM HEPES pH 7.5, 5.7 mM Tris-HCl pH 7.5, 10% glycerol, 62 mM l-proline, 1% EMPIGEN BB, 0.12 mM EDTA, and 113 mM KCl, and had a final volume of 12 μl. Binding reactions were carried out at 4°C for 1 h. Binding was resolved using 1% UltraPure agarose gel (Invitrogen) cast in a 15.7 cm × 10.1 cm tray, in 1× TBE. Gels were pre-run at 4°C for 45 min at 50 V, 10 μl of reactions were loaded, and samples were run at 4°C for 25 min at 150 V. For full-length NONO, binding reactions had a final buffer composition of 20 mM HEPES pH 7.4, 11 mM Tris-HCl pH 7.4, 105 mM KCl, 14% glycerol, 40 mM glycine, 0.2 mM EDTA, 0.5 mM DTT, 0.5% BSA and cOmplete, EDTA-free Protease Inhibitor Cocktail (Roche) in a final volume of 12 μl. Binding reactions were carried out at 25°C for 45 min. Binding was resolved using 0.8% UltraPure agarose gel (Invitrogen) in 1× TAE. Gels were pre-run at 4°C for 1 h at 80 V, 10 μl of reactions were loaded, and samples were run at 4°C for 50 min at 80 V. Gels containing (GGGGCC)_4_-Cy5 were imaged on the 700 nm channel of an Odyssey Infrared Imager (Li-Cor) and gels containing NEAT1_22619-Cy5 were imaged on the Cy5 channel of a Typhoon 9200 Variable Mode Imager (Molecular Dynamics).

For competition EMSAs all annealing was in the presence of potassium and labelled and unlabelled RNAs were annealed at 100 nM and 4 μM, respectively. 20 nM labeled RNA was pre-bound with 2.0 μM NONO(53-312) for 15 min at 4°C. Competitor RNA was added with indicated molar excess (relative to 10 nM labeled RNA) and labelled RNA and NONO(53-312) were, in turn, diluted to final concentrations of 10 nM and 1.0 μM. Reactions were incubated at 4°C for 1 h and resolved as described above.

Image adjustment and analysis was performed using ImageJ (NIH) (67). The Gel Analyzer tool was used to plot signal intensity by lane and signal from bound and unbound fractions was measured. Bound signal from the no-protein control was subtracted from bound signal of all lanes. Fraction bound was calculated and plotted against protein concentration or competitor molar excess. Curves were produced in Prism 5 (GraphPad Software, Inc.) by nonlinear regression with least squares fitting using the ‘One site - Total’ equation.

### Circular dichroism spectroscopy

RNA oligonucleotides were annealed and measured at 5 μM. Annealed RNA was measured in 1 mm pathlength quartz Suprasil cuvettes (Hellma Analytics) at 25°C with a Jasco J-810 Spectropolarimeter set as follows: scan: 320-220 nm; scanning mode: continuous; speed: 100 nm/min; response: 2 s; data pitch:1; accumulation: 5. Background signal from buffer was measured and subtracted from corresponding spectra. For TMPyP4 titration experiments settings were the same as above except for the following: speed: 200 nm/minute; accumulation: 3. Successively, 1 μl aliquots of 1 mM TMPyP4 stock were added and samples were incubated at room temperature for ten minutes before measuring.

### Cloning

The pCDNA3.1-(GGGGCC)_8_-eGFP, pCDNA3.1-(TGGTCC)_8_-eGFP, and pCDNA3.1-Spacer-eGFP plasmids were produced as follows. The eGFP coding sequence was amplified with forward primers producing an NheI site followed by the repeat sequence (GGGGCC)_8_, (TGGTCC)_8_, or ‘spacer’ sequence AGAGAATTCTGGAGGTGGCGGTTCT, and reverse primers producing an XhoI site. Amplified fragments were ligated into pCDNA3.1 via NheI and XhoI sites. The pCDNA3.0-eG-FP-tRSA and pCDNA3.0-eG-FP-tRSA-(GGGGCC)_70_ plasmids were produced as follows. The pcDNA3.0 backbone was linearized with XbaI and BamHI and a GFP-coding sequence containing an intron (‘eG-FP’), from pCA-G-intron-G (68) (gift from Liqun Luo, Addgene # 40027), and a 3′ EcoRI site was inserted using Gibson assembly to yield pCDNA3.0-eG-FP. A tRNA scaffolded SA ('tRSA') sequence was amplified from pcDNA3-tRSA (69) (gift from Ian Macara, Addgene # 32200) and ligated into the EcoRI site of pCDNA3.0-eG-FP to produce pcDNA3.0-eG-FP-tRSA. (GGGGCC)_70_ sequence flanked by EcoRI sites was cut from pCR8-70 (62) and inserted into the EcoRI site of pcDNA3.0-eG-FP-tRSA to produce pCDNA3.0-eG-FP-tRSA-(GGGGCC)_70_. For cloning and transfection plasmids were grown in NEB5α *E. coli* (New England Biolabs) at 30°C to minimize repeat instability and purified using standard procedures. Repeat insert orientation and size were confirmed by sequencing and agarose gel electrophoresis of EcoRI digestion products.

### Cell culture and transfection

HEK293T cells were cultured in Dulbecco's modification of Eagle's medium (DMEM) with glucose, l-glutamine, and sodium pyruvate (Corning) supplemented with 10% fetal bovine serum (gibco) and penicillin/streptomycin (gibco) at 37°C in 5% CO_2_. HEK293T cells were plated in 12-well plates at 8 × 10^5^ cells per well 24 h before transfecting. Lipofectamine 2000 (Invitrogen) was used to transfect 800 ng of plasmid per well following the manufacturer's protocol.

### RNA-immunoprecipitation

Antibody-bead conjugation was performed prior to harvesting cells. For each immunoprecipitation (IP), 2 μg of Mouse αNONO (Santa Cruz, sc-376865) or Mouse IgG isotype control (GeneTex, GTX35009) was conjugated to 33 μl Dynabeads Protein G (Invitrogen) suspension. Beads were washed in PBS-T (phosphate-buffered saline, 0.1% Tween-20) and then incubated with antibody diluted in PBS-T for ≥1 h at 4°C. Before addition of lysate, antibody-bead conjugates were washed for 5 min at 4°C once with PBS-T and then twice with RIP LBW buffer (50 mM Tris-HCl pH 7.5, 150 mM NaCl, 1% Triton X-100, 1 mM EDTA, 1 mM DTT).

For transfection experiments, lysate for each IP was collected from cells in a single well of a 12-well plate. For TMPyP4 titration experiments, stock lysate was produced from a 10 cm dish harvested at ∼80% confluency. To harvest, HEK293T cells were detached using TrypLE Express (gibco) and centrifuged at 300 g for 10 min to pellet. Pellets were rinsed with cold PBS, transferred to 15 ml Bioruptor Plus TPX tubes (Diagenode), and placed on ice until lysis. Pellets were resuspended in 1 ml RIP LBW buffer supplemented with cOmplete, EDTA-free Protease Inhibitor Cocktail (Roche). To lyse, resuspended cells were drawn into and forcefully expelled from 1 ml micropipette tips ten times and then incubated on ice for 30 min. In TMPyP4 titration experiments, lysate from each 10 cm dish was split into two tubes for sonication. Sonication was performed using a Bioruptor (Diagenode) with 15 ml tube adaptors. Two rounds of sonication were performed in ice cold water, each round consisting of 5 min of 15 s/90 s on/off cycles on low power. For TMPyP4 titration experiments protein content of stock lysate was measured by bicinchoninic acid (BCA) assay (Pierce) following the manufacturer's protocol. Stock lysate volume equivalent to 250 μg protein per IP was diluted in RIP LBW and TMPyP4 (Abcam) was added, giving a final volume of 1 ml. 5% of sample volume each was saved as ‘input’ for protein and RNA analyses. Lysate was applied to pre-conjugated beads and binding was carried out at 4°C for ∼16 h. Following IP, beads were washed for 5 min at 4°C four times with RIP LBW. Following washes, 5% sample volume of bead suspension was saved for protein and remaining beads were resuspended in Trizol (Invitrogen) and saved at -80°C until RNA purification.

### Reverse transcription and quantitative PCR

RNA extraction from RNA-IP samples was performed using Trizol according to manufacturer's protocol with the following modifications: RNA-IP samples in Trizol were passed ten times each through a 25G needle. Phase Lock Gel Heavy (5PRIME) was used to improve aqueous phase recovery, and GlycoBlue Coprecipitant (Ambion) was added to the aqueous phase prior to precipitation. Resulting pellets were resuspended in 30 μl DEPC-treated water.

cDNA was produced from 12 μl of RNA using the QuantiTect Reverse Transcription kit (Qiagen) according to manufacturer's protocol including a 30-minute 42°C incubation. qPCR was performed using iTaq Universal SYBR Green Supermix (BioRad) with a 10 μl reaction volume in a C1000 Touch thermal cycler with a CFX96 Real-Time System (BioRad). For assays measuring eGFP and NEAT1_2, 0.3 μl and 1 μl cDNA was added to each qPCR reaction, respectively. The qPCR protocol was as follows: (i) 95°C for 30 s; (ii) 95°C for 5 s; (iii) 60°C for 30 s + plate read; (iv) go to step (ii) 39 times. Primers for eGFP transcript detection were designed using Primer3, including eGFP 5′ Fwd-GACGTAAACGGCCACAAGTT and eGFP 5′ Rvs-AAGTCGTGCTGCTTCATGTG. Primers for NEAT1_2 were previously published (26), including NEAT1-2 Fwd-CAGTTAGTTTATCAGTTCTCCCATCCA, and NEAT1-2 Rvs-GTTGTTGTCGTCACCTTTCAACTCT.

### Western blot

Samples for western blotting were resolved on Criterion TGX 8-16% SDS-PAGE gels (BioRad). For RNA pulldowns, 25 μl of input (2.5% total input volume) and elutions were loaded. At room temperature, 80 V was applied for 1.5 h followed by 150 V for 30 min. For validation of RNA-IP, 20 μl of sample was loaded and 300 V was applied for 30 min on ice. Protein was transferred to nitrocellulose using a Trans-Blot Turbo Transfer System (BioRad) for 12 min at 2.5 A, 25 V and membranes were blocked with 5% BSA in PBS-T for 1 h at room temperature or overnight at 4°C. Primary antibodies, applied for 1 h at room temperature or overnight at 4°C, were diluted in blocking buffer as follows: Rabbit αNONO (Bethyl A300-587A) at 1:5,000; Rabbit αSFPQ (abcam ab38148) at 1:5,000; Rabbit αPSPC1 (Santa Cruz sc-84577) at 1:250; Rabbit αGAPDH (Pierce TAB1001) at 1:5,000; Mouse αHNRNPK (abcam ab39975) at 1:3,000. Secondary antibodies IRDye 800CW Goat αRabbit, IRDye 680LT Goat αRabbit, and IRDye 680LT Goat αMouse (Li-Cor), were diluted 1:10,000 in blocking buffer, applied for 1 h at room temperature, and imaged using an Odyssey Infrared Imager (Li-Cor). Between steps and prior to imaging, membranes were washed 3 times with PBS-T for 5-15 min at room temperature. PSPC1 staining was performed on membranes initially stained for NONO and hnRNP K and stripped with a 2× solution of NewBlot Nitro Stripping Buffer (Li-Cor) for 10 min at room temperature.

### RNA fluorescence *in situ* hybridization

10^5^ HEK293T cells were plated on polyethylenimine (PEI)-coated 12-mm coverslips (Deckgläser Cover Glass) and treated for 4 h with 0, 200, 400 and 500 μM TMPyP4 (Cayman Chemical, resuspended in water). Cells were washed with PBS, fixed with 3.7% formaldehyde in PBS for 10-15 min at room temperature, and permeablized with 70% ethanol at 4°C overnight. Following two washes in PBS and one wash in Wash Buffer (2× SSC, 10% deionized formamide), hybridization with 12.5 nM NEAT1_m probes (Biosearch Technologies, SMF-2037-1) in Hybridization Buffer (10% dextran sulfate, 10% deionized formamide, 2× SSC) was carried out for 4 h at 37°C. Following hybridization, coverslips were incubated in Wash Buffer for 30 min at 37°C, stained with DAPI diluted in Wash Buffer for 30 min at 37°C, and mounted with ProLong Gold Antifade (Thermo Fisher Scientific). Images were obtained using a Leica SP8 confocal microscope with 0.60 μM z-step size and matched exposure settings. Using ImageJ, images were thresholded and max projections were used to determine the mean number of foci per cell in each treatment.

### Bioinformatics

To identify putative G-quadruplex-forming sequences, the QGRS Mapper tool (http://bioinformatics.ramapo.edu/QGRS/index.php) (70) was used to analyze the full-length human NEAT1_2 sequence (NR_131012.1) with the following parameters: QGRS Max Length: 30, Min G-Group Size: 2, Loop size: from 0 to 36. For visualization, non-overlapping QGRS were mapped to human genome assembly hg19 and displayed as a custom track in UCSC Genome Browser (https://genome.ucsc.edu/) (71). The non-G-quadruplex form of the NEAT1_22619 oligonucleotide was predicted using the Mfold web server (http://www.bioinfo.rpi.edu/applications/mfold) (72). To determine QGRS enrichment relative to chance for each NEAT1_2 homologue (Mouse: NR_131212.1; Opossum: KX036208.1), five random sequences were generated with length and GC content equal to that of the homologue using the Random DNA Sequence Generator tool (https://faculty.ucr.edu/∼mmaduro/random.htm) and analyzed with QGRS Mapper.

Binding site data from enhanced crosslinking and immunoprecipitation (eCLIP) of NONO (ENCSR861PAR) and SFPQ (ENCSR965DLL) were downloaded from ENCODE (https://www.encodeproject.org/) (73). Plus-strand signals of unique NONO eCLIP reads were obtained in bigWig format (replicate 1: ENCFF715MPQ; replicate 2: ENCFF043RMQ; NONO eCLIP mock input: ENCFF187ARS). NONO eCLIP peaks were obtained in narrowPeak bed and bigBed format for replicates and merged peaks, respectively (replicate 1: ENCFF918YLZ; replicate 2: ENCFF444YFD; merged: ENCFF730QRI). eCLIP reads and peaks were visualized using the Integrative Genomics Viewer (https://software.broadinstitute.org/software/igv/) (74). Data processing and analysis was performed on Ubuntu using the Windows Linux bash shell. File conversion from bigBed to bed was performed with the UCSC bigBedToBed utility. Prior to statistical analysis, peaks were limited to those mapped within the NEAT1 genomic locus using the awk command. The Fisher's exact test was performed using the fisher utility from the bedtools suite v2.29 (https://github.com/arq5x/bedtools2/releases) (75) to compare NEAT1 non-overlapping QGRS to NONO eCLIP peaks, and reported *P*-values are from two-tailed analysis. To account for pre-analysis limitation of eCLIP peaks to only those within the NEAT1 gene, the ‘genome’ size used for fisher analysis was the size of the NEAT1 gene.

## RESULTS

### NONO recognizes G-quadruplex structures formed by G-rich repeat RNA

**Figure 1.**
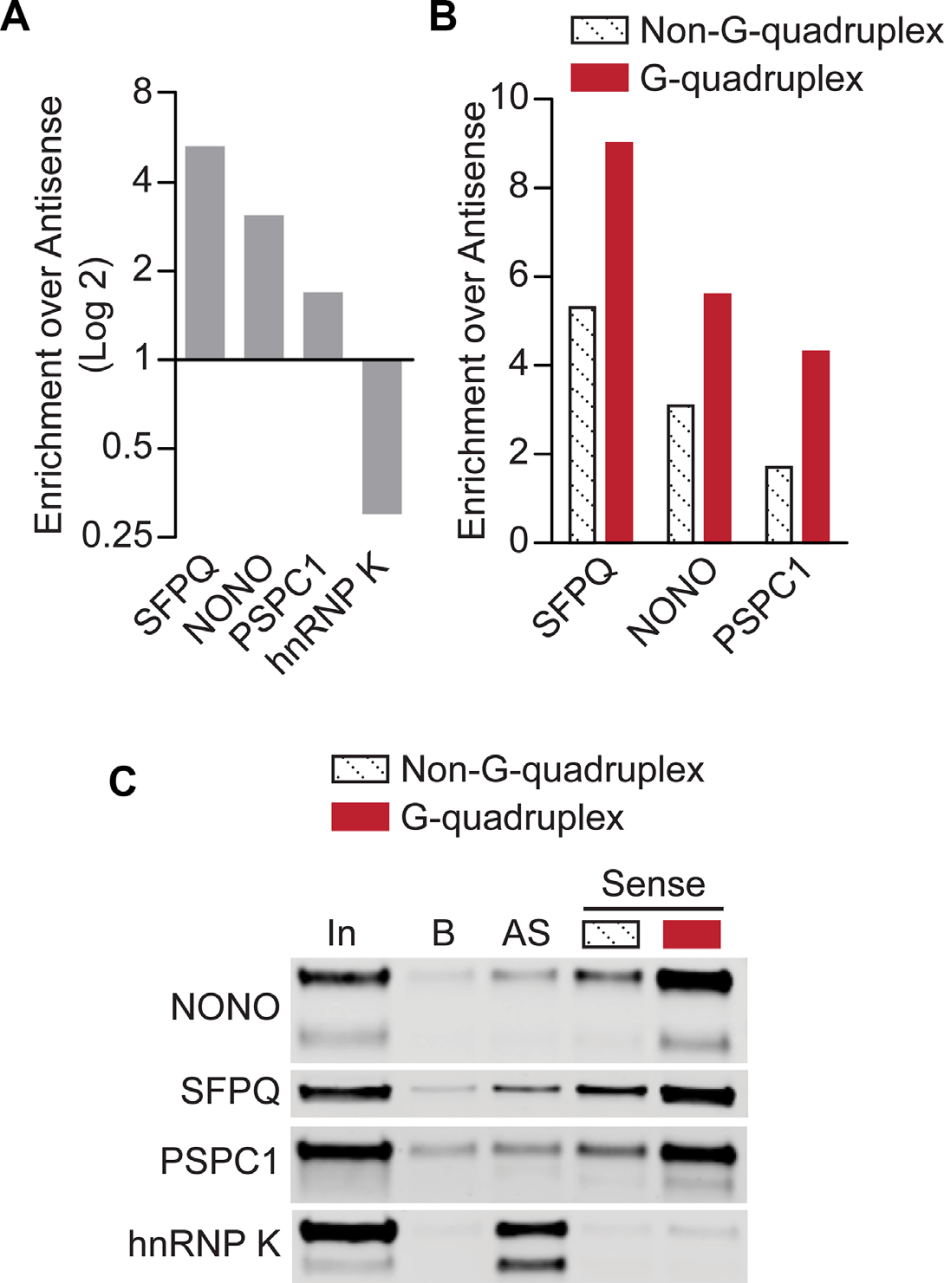
DBHS proteins are preferentially enriched by GGGGCC repeat RNA in the G-quadruplex conformation. (**A**) Comparative enrichment of DBHS-family proteins and hnRNP K by (GGGGCC)_4_ RNA and (CCCCGG)_4_ RNA. SILAC was used for comparative analysis of proteins enriched by pulldown with biotinylated RNA oligonucleotides from HEK293T lysate (62). Enrichment by G-rich ‘Sense’ relative to enrichment by C-rich ‘Antisense’, as indicated by SILAC ratios of (GGGGCC)_4_ / (CCCCGG)_4_, is plotted; a log2 scale is used to illustrate strand preference. (**B**) Comparative enrichment of DBHS-family proteins by (GGGGCC)_4_ RNA in G-quadruplex and non-G-quadruplex conformations, shown relative to enrichment by (CCCCGG)_4_ RNA. SILAC ratios for (GGGGCC)_4_/(CCCCGG)_4_ are shown for both (GGGGCC)_4_ conformations. (**C**) Western blot validation of RNA pulldown results. 2.5% input volume was loaded for reference (‘In’). Pulldown with bead-only control (‘B’) was compared to antisense (‘AS’; (CCCCGG)_4_) RNA and ‘Sense’ ((GGGGCC)_4_) RNA in non-G-quadruplex and G-quadruplex conformations. Results shown are representative of three replicates.

**Figure 2.**
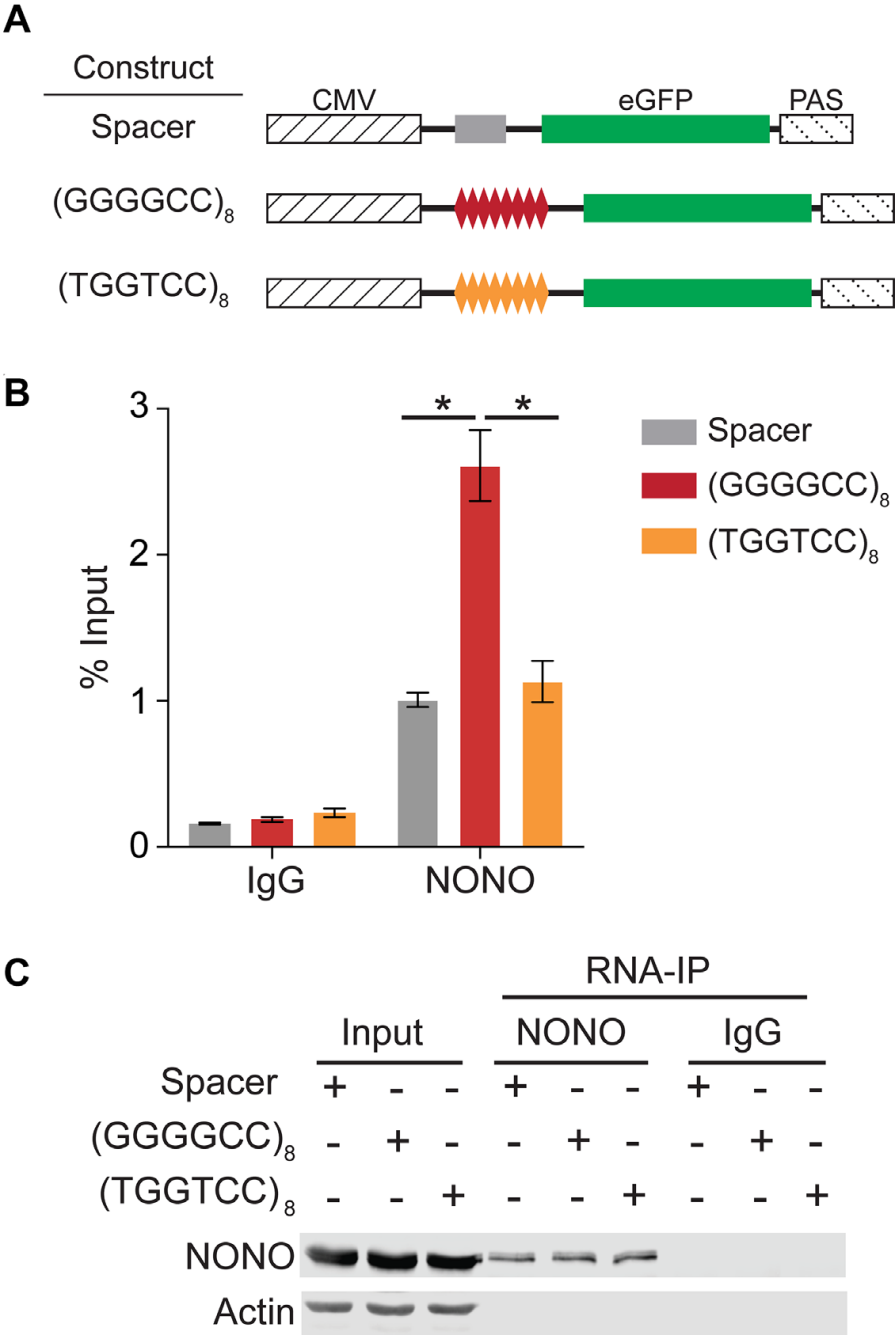
GGGGCC repeats recruit NONO to model transcripts. (**A**) An eGFP-encoding model transcript system was used to test whether the presence of GGGGCC repeat sequence affects NONO-transcript association in a cellular context. Constructs contained an eGFP coding sequence transcribed under the control of a cytomegalovirus (CMV) promoter and bovine growth hormone polyadenylation signal (PAS), with experimental sequences inserted in the 5′-untranslated region. Two control transcripts were used: the Spacer construct included a 25-nucleotide spacer insert and the (TGGTCC)_8_ construct included eight TGGTCC repeats, providing a mutated repeat sequence incapable of forming G-quadruplex structures. (**B**) RNA-IP for NONO was performed on HEK293T cells transfected with the model transcript constructs, followed by RT-qPCR to compare enrichment of the model transcripts. Transcript enrichment is expressed as percentage of input with SEM (*n* = 3). ‘*’ indicates *P* < 0.05 (two-tailed *t*-test). (**C**) Western blot of NONO IP fractions, 5% input, and enriched fraction from IP with IgG control. Actin is blotted as a negative control for IP enrichment.

*C9orf72* hexanucleotide repeat RNA has been linked to neurodegeneration and significant effort has gone into identifying protein interactors (62,76–79). In a previous study we performed a pulldown with repeat RNA of the sense (GGGGCC) and antisense (CCCCGG) sequences and used the mass spectrometry technique Stable Isotope Labelling by Amino acids in Cell culture (SILAC) to determine relative enrichment (62). We noted that all three members of the DBHS family, NONO, SFPQ, and PSPC1, were preferentially enriched by the G-rich sense RNA (Figure 1A). In part, this finding was consistent with previous studies demonstrating G-rich RNA binding activity of NONO (80–82). As a control, the RNA-binding protein hnRNP K, known to interact with C-rich RNA (83), was examined and found to be preferentially enriched by the C-rich antisense RNA (Figure 1A). In addition to comparing proteins enriched by sense and antisense RNA pulldowns, we used SILAC to compare enrichment by pulldown with sense RNA in the G-quadruplex and non-G-quadruplex conformations (62). The non-G-quadruplex form of the sense repeat sequence GGGGCC assumes a hairpin conformation (62). All three members of the DBHS protein family showed preferential enrichment by the G-quadruplex conformation (Figure 1B). SILAC findings for enrichment of NONO, SFPQ, PSPC1, and hnRNP K by repeat RNA were validated by western blotting (Figure 1C). To test the effect of GGGGCC repeats on NONO recruitment in cells, we inserted repeats into the terminal untranslated region of a model eGFP transcript and measured NONO-transcript association by RNA immunoprecipitation (RNA-IP). Consistent with the known affinity of NONO for G-rich RNA *in vitro* (62,80–82), we found that inclusion of GGGGCC repeats increases association of NONO with the model transcript (Figure 2). Preventing G-quadruplex formation using a G-tract mutation approach (51) abolished this increased association, consistent with the structural preference we observed *in vitro*. Inclusion of a longer GGGGCC repeat sequence was tested in the context of an alternative model transcript system and also resulted in increased association with NONO (Supplementary Figure S1).

**Figure 3.**
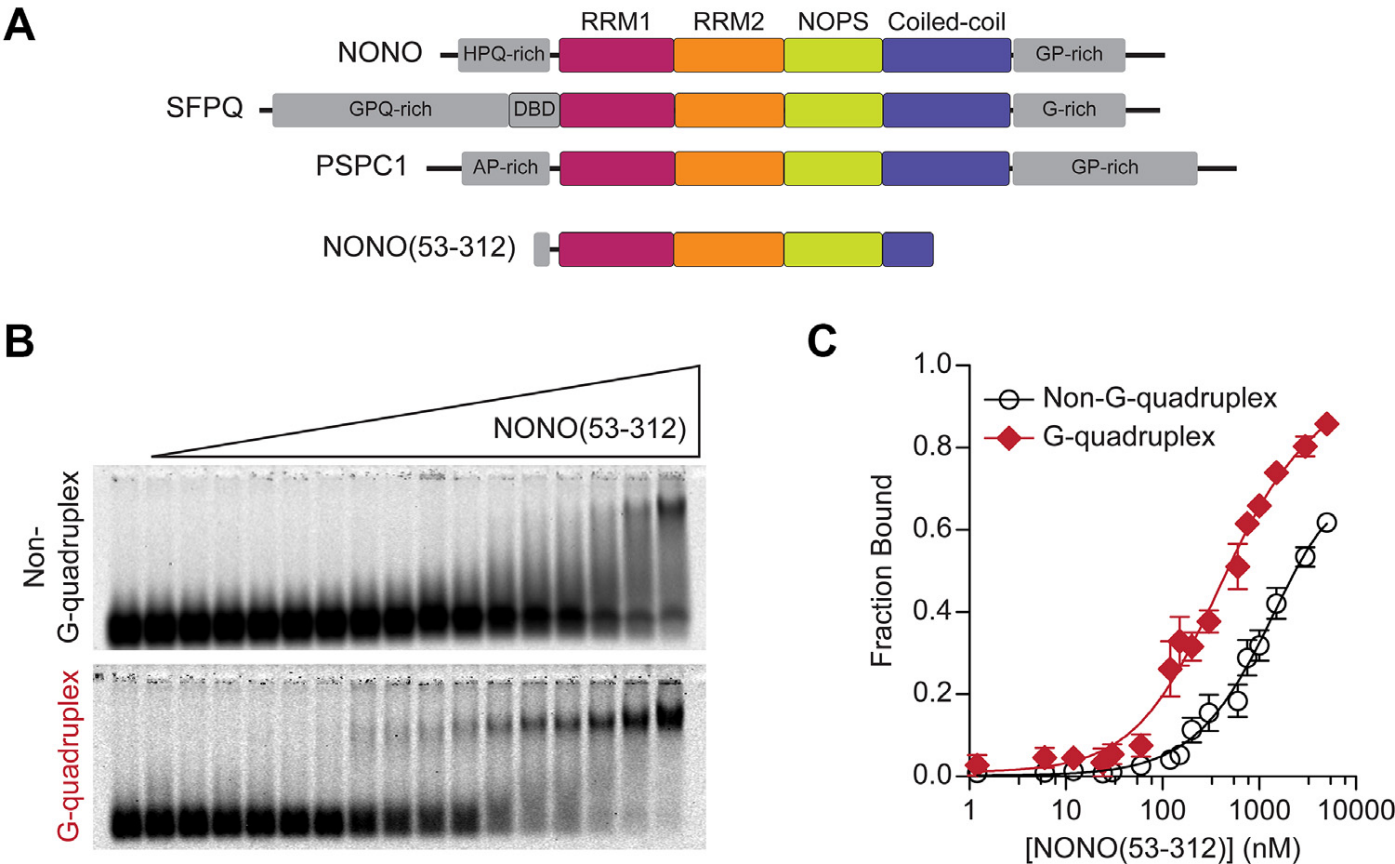
NONO preferentially binds GGGGCC repeat RNA in the G-quadruplex conformation. (**A**) Domain architecture of DBHS proteins and NONO(53-312) construct with shared DBHS core region colored by domain and low-complexity regions in grey. (**B**) EMSA with recombinant NONO(53-312) and (GGGGCC)_4_ RNA in G-quadruplex and non-G-quadruplex conformations. 10 nM labelled (GGGGCC)_4_ RNA was incubated with NONO(53-312) at the following concentrations (left to right): 0, 1.2 nM, 6.0 nM, 12 nM, 24 nM, 30 nM, 60 nM, 120 nM, 150 nM, 200 nM, 300 nM, 600 nM, 750 nM, 1.0 μM, 1.5 μM, 3.0 μM, 5.0 μM. For visualization purposes, images were adjusted independently to account for differences in fluorescence intensity between conformations. Quantification was performed prior to this adjustment. (**C**) Data fit of (B) with binding curves and SEM (*n* = 3). In this and subsequent figures, error bars for some data points are within the size of the symbol and are therefore not visible.

Exploring the possibility that NONO RNA binding specificity goes beyond reported sequence preferences, we next sought to directly test structural discrimination using electrophoretic mobility shift assays (EMSAs) with purified recombinant NONO. NONO consists of 471 amino acids including an amino-terminal histidine/proline/glutamine-rich region, a central DBHS region, and a carboxyl-terminal glycine/proline-rich region (Figure 3A) (84). The low-complexity N- and C-terminal regions contain functional sequences including nuclear localization signals and are predicted to be disordered (85,86). The central DBHS region, which is conserved among members of the DBHS family, contains two RRMs, a protein-protein interaction motif unique to DBHS proteins known as the NONA/paraspeckle (NOPS) domain, and a coiled-coil domain (84). Previous efforts to produce recombinant full-length NONO have been impeded by solubility issues, and so for *in vitro* experiments we purified a major portion of NONO containing the central DBHS region, which has been optimized for recombinant expression and purification and characterized in prior structural studies (66,85,87). The purified protein, NONO(53-312), consists of both RRMs, the NOPS domain, and approximately half of the coiled-coil domain (Figure 3A, *bottom*). An RNA oligonucleotide consisting of four *C9orf72* sense-strand repeats, denoted ‘(GGGGCC)_4_’, was annealed in the absence or presence of 100 mM KCl to induce non-G-quadruplex or G-quadruplex conformations, respectively, as previously described (62). Labelled (GGGGCC)_4_ RNA was incubated with increasing concentrations of NONO(53-312), binding was resolved by EMSA (Figure 3B), and complex formation was quantified and plotted for comparison (Figure 3C). (GGGGCC)_4_ RNA in the G-quadruplex conformation formed complexes with NONO(53-312) at lower concentrations than RNA in the non-G-quadruplex conformation, indicating preferential binding of NONO to the G-quadruplex conformation.

### NEAT1 contains abundant G-quadruplex motifs

**Figure 4.**
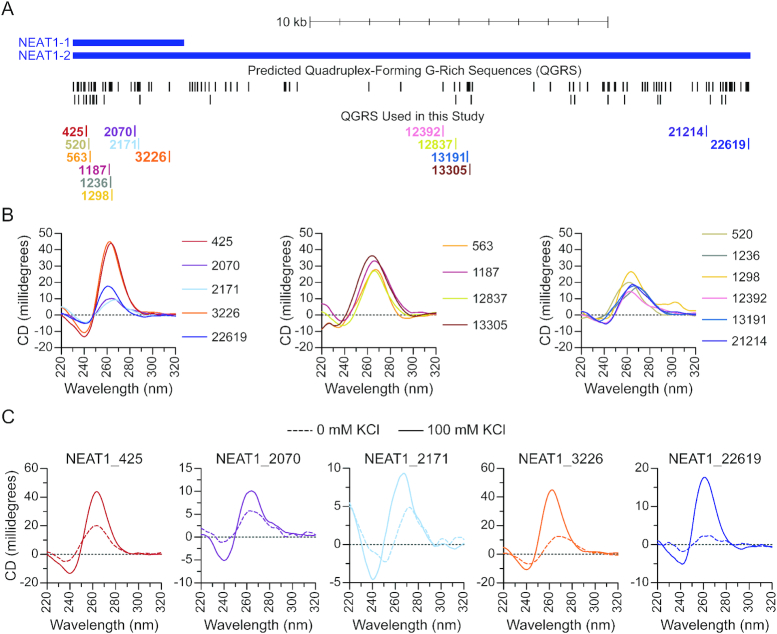
NEAT1 contains abundant G-quadruplex-forming sequences. (**A**) Quadruplex-forming G-rich sequences (QGRS) were predicted along the length of NEAT1 using QGRS Mapper. Predicted QGRS are shown mapped to NEAT1 (upper track). The subset of QGRS used in this study are identified by starting nucleotide position relative to the 5′ end of NEAT1 (lower track). (**B**) Circular dichroism spectra of NEAT1 G-rich RNAs annealed in the presence of 100 mM KCl, grouped as follows: QGRS with higher G-scores (left) and lower G-scores (center and right). (**C**) Circular dichroism spectra of NEAT1 G-rich RNAs with highest G-scores annealed in the presence (solid line) and absence (dashed line) of 100 mM KCl.

Association of NONO with the lncRNA NEAT1 is a critical step in paraspeckle assembly (18,23–25,31), but the specific elements of NEAT1 mediating the interaction have yet to be defined (34–36). NEAT1 has relatively low sequence conservation (32), and it has been speculated that structural elements, rather than particular sequence motifs, may facilitate protein recruitment (34,36,38). Our finding that NONO recognizes G-quadruplex structures formed by GGGGCC repeat RNA led us to wonder whether G-quadruplex recognition could be involved in recruitment to NEAT1. To explore this possibility, we first sought to determine whether NEAT1 contains sequences capable of forming G-quadruplexes. Several factors determine the propensity of a given sequence for G-quadruplex formation, including the number and length of G-tracts as well as the length and nucleotide content of intervening sequences (39,88). Using the quadruplex-forming G-rich sequences (QGRS) Mapper tool (70), we determined that NEAT1 contains segments with characteristics favoring G-quadruplex formation, yielding 111 non-overlapping QGRS (Figure 4A). The predicted QGRS are distributed along the length of NEAT1, with notable clusters near each end as well as in several internal positions. We also compared their G-scores, a metric calculated by QGRS Mapper, to predict the relative propensity of the sequences to form G-quadruplexes. Based on G-score and location relative to known functional domains, we selected a series of putative G-quadruplex-forming sequences for further study, each identified by its nucleotide position relative to the 5′ end of NEAT1 (Figure 4A, *lower track*; Table 1).

**Table 1.** Oligonucleotide sequences used in this study. Sequences with a G-score of ‘N/A’ are not predicted to form G-quadruplexes.

Name	Sequence	G-score
NEAT1_425	GGGUGCAGGGCUGCAGGAGGGCCGGGAGGG	38
NEAT1_520	GGGGAUGAGGCCUGGUCUUGUGG	18
NEAT1_563	GGCUGACCGCUCUGGCCCAGGGUGG	12
NEAT1_1187	GGAACCGGGAGACAUGGAGUCCCUGG	19
NEAT1_1236	GGAGGGGAGACCCUGGAGG	15
NEAT1_1298	GGCUUAGGAGGAGG	18
NEAT1_2070	GGGAGUUGUGGGGGACGGGGAGAAGGGG	41
NEAT1_2171	GGGAUGAGGGUGAAGAAGGGGAGAGGG	39
NEAT1_3226	GGGAAGGGGAUGGGGAUUGUGGG	40
NEAT1_12392	GGAUGGGGCUGUGG	17
NEAT1_12837	GGCUCUUGGCACAUCUGGUUGG	16
NEAT1_13191	GGCAUCCUGGUCUGGGAAGG	18
NEAT1_13305	GGAGCUCAGGUGUGAGCCCGGCUCUGG	16
NEAT1_21214	GGCAGGUUGGGACUUAGAUGG	15
NEAT1_22619	GGGAGGGAGGGAGGG	42
(GGGGCC)_4_	GGGGCCGGGGCCGGGGCCGGGGCC	63
Scrambled	GCGAGUGUGUGAGACGUCGC	N/A
5′-Splice Site	AAAAAGGUAAG	N/A

Next, we used circular dichroism (CD) spectroscopy to assay the secondary structures of the selected sequences from NEAT1. The parallel G-quadruplex topology typically assumed by RNA displays characteristic spectral features including a peak near 260 nm and trough near 240 nm (41,89). To determine whether selected NEAT1 sequences form G-quadruplexes, we first performed a screen of the 15 selected RNAs under conditions favorable to G-quadruplex formation. CD spectra of these oligonucleotides annealed in the presence of potassium are overall characteristic of G-quadruplex RNA (Figure 4B). Oligonucleotides with the highest G-scores show distinct peaks near 260 nm and troughs near 240 nm, except for NEAT1_2171 which shows a main peak near 270 nm (Figure 4B, *left*). Spectra from sequences with lower G-scores were more diverse and were qualitatively grouped into two subsets. Each in the first subset has a single broad peak near 260 nm and trough near 240 nm (Figure 4B, *middle*). In the second subset (Figure 4B, *right*) all show main peaks near 260 nm except for NEAT1_21214, with a main peak above 270 nm. All in this group display a hip component to the main peak and/or a distinguishable secondary peak, features which suggest the presence of a conformationally mixed population (90). Five sequences with the highest G-scores were chosen for further characterization: NEAT1_425, NEAT1_2070, NEAT1_2171, NEAT1_3226 and NEAT1_22619. RNA molecules containing the sequence characteristics required to form monomeric intramolecular G-quadruplexes have not, to the best of our knowledge, been found to produce multi-strand intermolecular G-quadruplexes, retaining their intramolecular G-quadruplex form at concentrations much higher than those used in this work (91,92). Accordingly, the oligonucleotides examined here are unlikely to form intermolecular G-quadruplexes. Stabilization by potassium is a well-defined property of many G-quadruplexes, therefore a shift towards the characteristic spectral signatures described above upon inclusion of potassium cations is an informative indicator of G-quadruplex structure (89). Accordingly, effects of potassium addition were determined for the selected sequences (Figure 4C). For all sequences tested, the addition of potassium caused a shift toward G-quadruplex spectral signatures. The most dramatic change observed was in NEAT1_22619, where a nearly flat spectrum indicates little G-quadruplex structure in the absence of potassium. Absent Watson-Crick-pairable bases and confirmed by analysis with Mfold to lack possible alternative secondary structures, NEAT1_22619 is expected to assume the form of a flexible linear polymer resembling that of previously characterized unstructured RNAs (93) in the absence of the G-quadruplex fold.

### NONO recognizes NEAT1 G-quadruplexes

**Figure 5.**
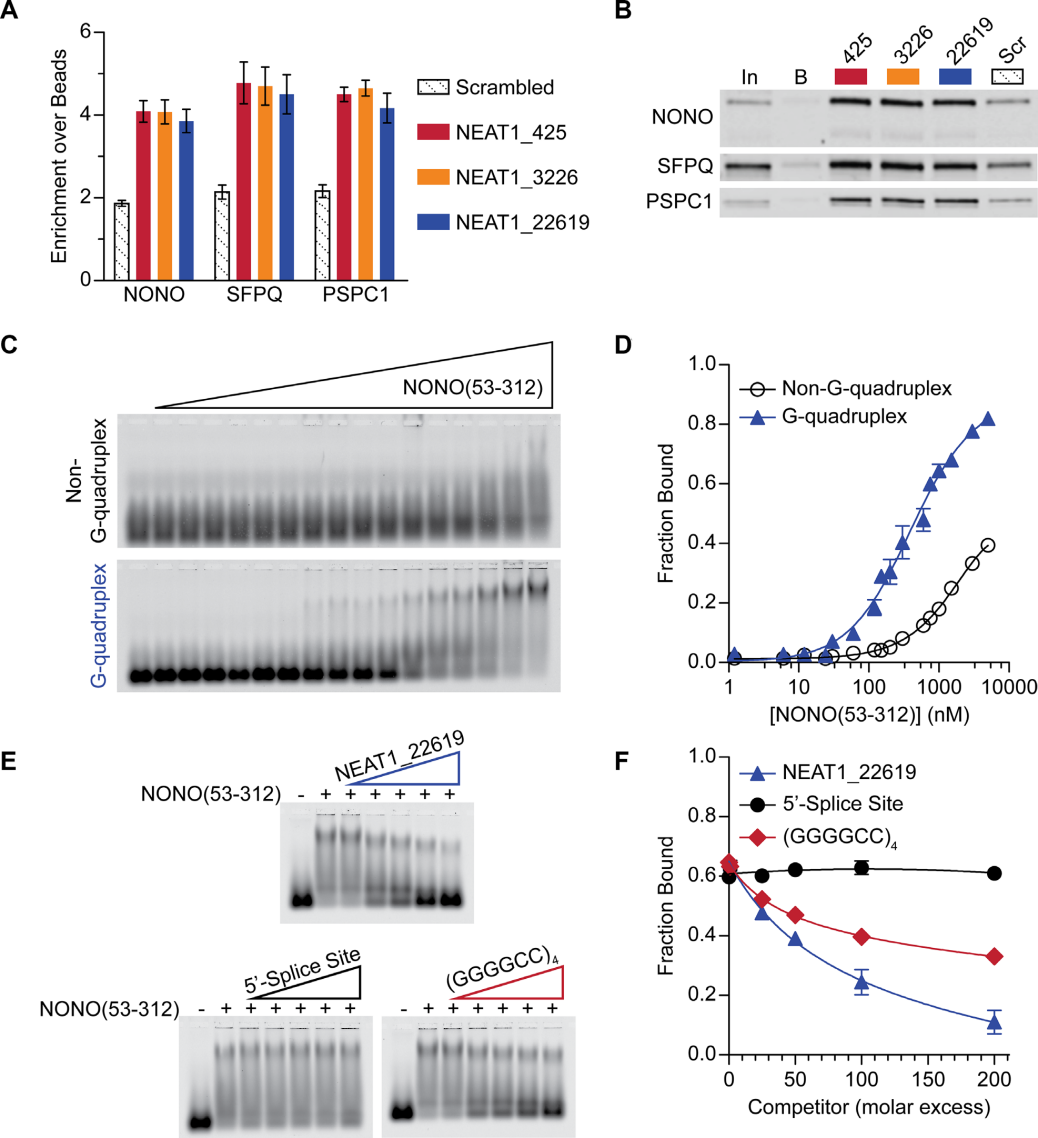
NONO preferentially interacts with NEAT1 G-rich RNA in the G-quadruplex conformation. (**A**) Comparative enrichment of DBHS-family proteins by NEAT1 G-quadruplex RNA and non-G-quadruplex-forming G-rich RNA ‘Scrambled’. Mass spectrometry with TMT labelling was used for comparative analysis of proteins enriched from HEK293T lysate by pulldown with biotinylated RNA oligonucleotides. Enrichment is shown as mean and SEM of RNA-enriched abundance, relative to enrichment by beads, of each protein's peptides. (**B**) Western blot validation of RNA pulldown results. 2.5% input volume (‘In’) was loaded for reference. Pulldown with bead-only control (‘B’) was compared to NEAT1 G-quadruplex RNAs and non-G-quadruplex-forming G-rich (‘Scr’) RNA. Results shown are representative of three replicates. (**C**) EMSA with recombinant NONO(53-312) and NEAT1_22619 RNA in G-quadruplex and non-G-quadruplex conformations. 10 nM labelled NEAT1_22619 RNA was incubated with NONO(53-312) at the following concentrations (left to right): 0, 1.2 nM, 6.0 nM, 12 nM, 24 nM, 30 nM, 60 nM, 120 nM, 150 nM, 200 nM, 300 nM, 600 nM, 750 nM, 1.0 μM, 1.5 μM, 3.0 μM, 5.0 μM. (**D**) Data fit of (C) with binding curves and SEM (*n* = 4). (**E**) Competition EMSA with recombinant NONO(53-312) and labelled NEAT1_22619 G-quadruplex. Labelled RNA only and labelled RNA with NONO(53-312) but no competitor are shown in lanes 1 and 2, respectively. Labelled NEAT1_22619 was pre-incubated with NONO(53-312) before addition of unlabelled competitor RNAs at 1-200× molar excess (lanes 3-7) relative to 10 nM labelled RNA. When present, NONO(53-312) was at 1.0 μM. Unlabelled competitors include NEAT1_22619 RNA in G-quadruplex conformation (upper), 5′-Splice Site RNA known to bind NONO via RRM1 (lower left), and (GGGGCC)_4_RNA in G-quadruplex conformation (lower right). All competitors were pre-annealed with 100 mM KCl. (**F**) Data fit of (E) with binding curves and SEM (*n* = 3).

We next performed a pulldown experiment to explore the association of native paraspeckle proteins with a series of NEAT1 G-quadruplexes. Using mass spectrometry with quantitative comparisons through tandem mass tag (TMT) labeling, we found that a notable selection of paraspeckle proteins were enriched by NEAT1 G-quadruplex RNA (Supplementary Figure S2, Supplementary Table S1). NONO, SFPQ, and PSPC1 were found to be enriched by NEAT1 G-quadruplexes (Figure 5A) and this enrichment was validated by western blotting (Figure 5B). Having identified the ability of NONO to discriminate between structures of GGGGCC repeat RNA and finding NONO among proteins enriched by pulldown with NEAT1 G-quadruplexes, we wondered whether NONO could discriminate between G-quadruplex and non-G-quadruplex structures of NEAT1 G-rich RNA. To test this possibility, labelled, alternatively annealed RNA was incubated with increasing concentrations of protein and then binding was resolved by EMSA. Having displayed a robust and clearly inducible G-quadruplex conformation during initial characterization, the NEAT1_22619 RNA was selected for use in gel shift experiments. Initial attempts using commercially sourced recombinant full-length NONO found the G-quadruplex conformation to shift with lower concentrations of NONO than the non-G-quadruplex conformation, suggesting that full-length NONO shows preferential binding to the G-quadruplex conformation (Supplementary Figure S3). A large portion of RNA shifting with full-length NONO was retained at signal-saturating levels in wells, resembling that seen in a previously published experiment interrogating SFPQ association with DNA (33), and presumably a product of extended coiled-coil oligomerization domains shared by both SFPQ and NONO. To obtain quantifiable results, NONO(53-312) was used in subsequent experiments. Binding of NONO(53-312) to alternatively annealed NEAT1_22619 RNA was resolved by EMSA (Figure 5C), and complex formation was quantified and plotted for comparison (Figure 5D). Consistent with our findings for G-rich repeat RNA, NEAT1_22619 RNA in the G-quadruplex conformation was found to shift with lower concentrations of NONO(53-312) compared to the non-G-quadruplex conformation, indicating preferential binding to the G-quadruplex conformation. To test the specificity of the NONO-NEAT1_22619 G-quadruplex interaction, a series of competition EMSAs were performed in which pre-bound NONO(53-312)-NEAT1_22619 G-quadruplex was incubated with unlabelled competitor RNA at increasing molar excess (Figure 5E). Results were quantified and fraction bound was plotted (Figure 5F). Competition with excess unlabelled NEAT1_22619 G-quadruplex reduced binding of labeled RNA to a small fraction of that bound in the absence of the competitor (Figure 5E, *upper*). 5′ Splice Site RNA, an AU-rich RNA known to bind the RRM1 domain of NONO (80,94), did not compete with the NEAT1_22619 G-quadruplex to a significant extent, supporting the specificity of the NONO(53-312)-NEAT1_22619 G-quadruplex interaction (Figure 5E, *lower left*). Competition with (GGGGCC)_4_ G-quadruplex significantly reduced the fraction of NEAT1_22619 bound, indicating that GGGGCC repeat RNA G-quadruplexes can compete with NEAT1_22619 G-quadruplexes for binding to NONO(53-312) (Figure 5E, *lower right*).

### G-quadruplex motifs mediate NONO-NEAT1 association

**Figure 6.**
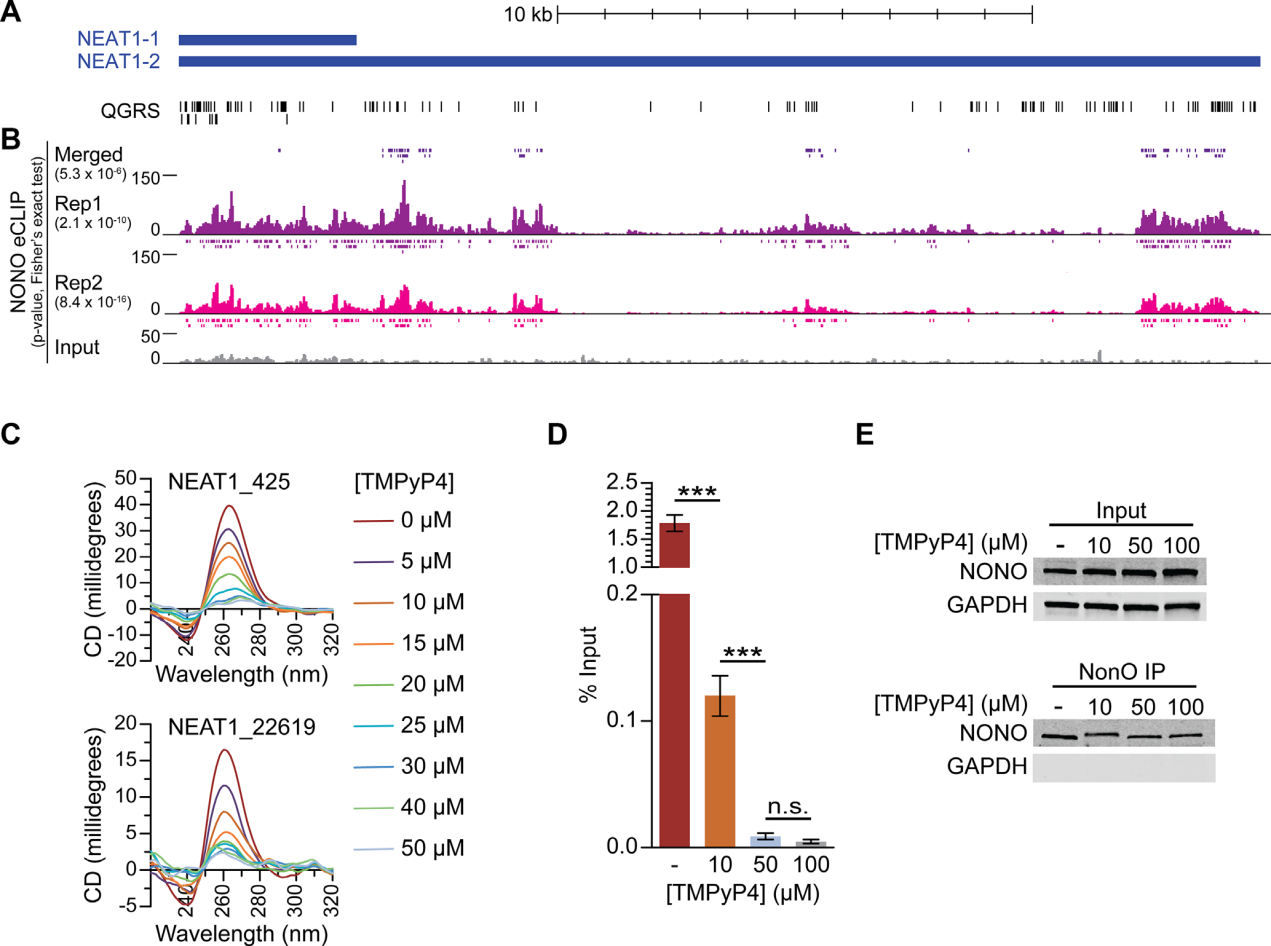
NONO binds to NEAT1 through G-quadruplex motifs. (**A**) Quadruplex-forming G-rich sequences (QGRS) mapped to NEAT1 are shown as tall bars (black). (**B**) NONO eCLIP data mapped to NEAT1. Reads are plotted by intensity and called peaks are shown as short bars. Tracks, from top to bottom: Merged peaks, determined by replication and adjustment for input (dark purple); Replicate 1 read intensity plot with called peaks directly beneath (purple); Replicate 2 read intensity plot with called peaks directly beneath (pink); size-matched paired input read intensity plot (grey). The statistical significance of overlap between QGRS and each set of peaks was determined using the two-tailed Fisher's exact test, with *P*-values shown in parentheses beneath each corresponding track label. (**C**) Circular dichroism of 5 μM NEAT1_425 and NEAT1_22619 G-quadruplex RNAs following addition of TMPyP4 at increasing concentrations. (**D**) NEAT1 enrichment by NONO RNA-IP with addition of TMPyP4. RNA-IP for NONO was performed on HEK293T cell lysate. Various concentrations of TMPyP4 were added to lysate before immunoprecipitation. RT-qPCR was performed to measure NEAT1 enrichment, expressed as percentage of input (*n* = 4). '***' indicates *P* < 0.0005; 'n.s.' indicates *P* ≥ 0.05 (two-tailed *t*-test). (**E**) Western blot of RNA-IP samples described in (D) and 5% input. GAPDH was blotted as a negative control for IP enrichment.

Having found that NEAT1 contains abundant G-quadruplex motifs and that NONO binds NEAT1 sequences with preference for the G-quadruplex conformation, we sought to determine whether the recognition of and preference for NEAT1 G-quadruplexes observed *in vitro* is reflected in living cells. Enhanced crosslinking and immunoprecipitation (eCLIP), which allows for identification of *in vivo* binding sites with single-nucleotide level resolution, was used to identify NONO binding sites in the human K562 cell line as part of the ENCODE project (73). Examining these data, we observed enrichment of NONO in multiple regions across the length of NEAT1 and, to a large extent, these regions appeared to overlap with G-quadruplex-forming segments (Figure 6A,B). Analysis by Fisher's exact test revealed that the extent of overlap with NEAT1 G-quadruplex-forming sequences was highly statistically significant for both eCLIP replicates as well as for merged peaks determined by replication and adjustment for input (Figure 6B). While a portion of NONO eCLIP sites may reflect binding of NONO/SFPQ heterodimers, overlap of SFPQ eCLIP binding sites with NEAT1 G-quadruplex-forming segments was not statistically significant (Supplementary Figure S4), indicating that SFPQ binds to NEAT1 through additional or alternative means which do not account for the specificities revealed by NONO eCLIP. It is important to note that an unknown fraction of reads mapped to the 5′ end of NEAT1 reflect interactions with the short isoform of NEAT1, although sites overlapping with G-quadruplex motifs on either isoform are relevant to NONO binding specificity. Strikingly, NONO-bound sites present in the adjusted and replicated ‘Merged’ set are nearly exclusive to the NEAT1_2 isoform. The extent of NONO overlap with G-quadruplex-forming segments is well beyond that expected by chance, providing evidence that the NONO preference for G-quadruplex RNA observed *in vitro* also applies to native NONO in living cells. Furthermore, these results indicate that NONO binds to NEAT1 *in vivo* largely through G-quadruplex-forming sequences, suggesting that this binding activity may mediate critical NONO-NEAT1 interactions.

The cationic porphyrin tetra-(*N*-methyl-4-pyridyl)porphyrin (TMPyP4), known to bind and distort previously described RNA G-quadruplexes, is commonly used to study protein–G-quadruplex interactions (95–97). To determine whether this drug has similar effects on NEAT1 G-quadruplexes, we measured the circular dichroism of select NEAT1 G-quadruplexes upon addition of increasing concentrations of TMPyP4. For both NEAT1_425 and NEAT1_22619 RNAs, spectra showed concentration-dependent decreases at 260 nm and increases at 240 nm, indicating loss of G-quadruplex structure (Figure 6C).

With eCLIP revealing significant overlap of NONO-bound sites and NEAT1 G-quadruplex motifs, we wondered whether G-quadruplex structures are critical to NONO-NEAT1 association. Observing a decrease in NEAT1 foci upon treating HEK293T cells with TMPyP4 (Supplementary Figure S5), we sought to determine whether disruption of NEAT1 G-quadruplexes by TMPyP4 would affect association with NONO. To study this association using native full-length molecules, RNPs were isolated from cell lysate by RNA-IP. Prior to immunoprecipitation, TMPyP4 was added to HEK293T lysate at increasing concentrations. TMPyP4 was added post-lysis to avoid potential confounding effects at the level of gene expression. Following immunoprecipitation of NONO, NEAT1 enrichment was measured by RT-qPCR (Figure 6D). TMPyP4 was found to cause dramatic dose-dependent decreases in isolated NEAT1, suggesting that G-quadruplexes play a significant role in NONO-NEAT1 association. Western blotting indicated that NONO input and enrichment by IP was consistent among treatments, confirming that the changes observed by RT-qPCR were due to altered NONO-NEAT1 association (Figure 6E).

### Enrichment in G-quadruplex motifs is a conserved feature of NEAT1 homologues

**Figure 7.**
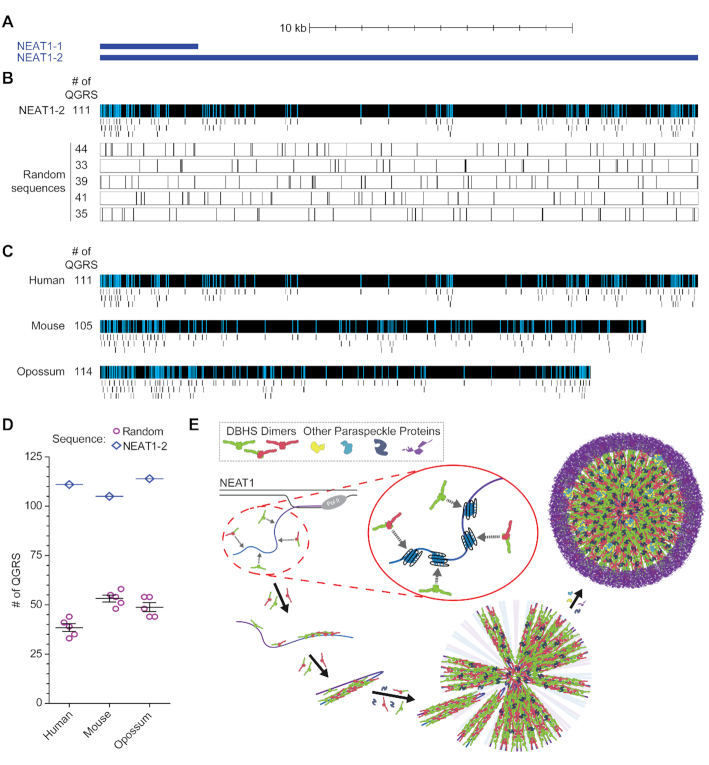
Enrichment in G-quadruplex motifs is a conserved feature of NEAT1 homologues. (**A**) Human NEAT1 isoforms and scale bar. (**B**) Quadruplex-forming G-rich sequences (QGRS) on human NEAT1 (upper track) and on randomly generated sequences (lower tracks). QGRS are shown mapped to NEAT1 (upper track, light blue bars) and expanded below (upper track, short black bars). QGRS (tall black bars) were identified for five randomly generated sequences with length and GC content equal to NEAT1 (lower tracks). The number of QGRS identified on each sequence is shown to the left of the corresponding track. (**C**) Quadruplex-forming G-rich sequences predicted along the length of NEAT1 homologues. Identified QGRS are shown mapped to each NEAT1 homologue (light blue bars) and expanded below (short black bars). (**D**) QGRS enrichment among NEAT1 homologues. Sequences were randomly generated with the length and GC content of NEAT1 homologues and QGRS were identified. For each homologue, numbers of QGRS on five generated sequences are plotted with mean and SEM, with the number of QGRS on the corresponding homologue plotted for comparison. (**E**) Paraspeckle assembly begins with co-transcriptional recruitment of DBHS dimers (NONO and SFPQ homo- and heterodimers) to NEAT1, seeding formation of NEAT1 RNPs, the primary subunit of paraspeckles, and initiating paraspeckle formation. The elements of NEAT1 responsible for this recruitment have yet to be identified. G-quadruplex structures along the length of NEAT1 are recognized by NONO with structural specificity, play a critical role in NONO-NEAT1 association, and are a conserved feature among NEAT1 homologues. These findings position NEAT1 G-quadruplexes as a primary candidate for the NONO-recruiting elements of NEAT1.

The specific elements of NEAT1 mediating interaction with DBHS proteins have remained largely undefined (34–36). Given the low sequence conservation of NEAT1 (32), it has been speculated that structural elements, rather than particular sequences, may facilitate recruitment of these proteins (34,36,38). Our data suggest that G-quadruplex motifs may be a structural element of NEAT1 involved in recruiting NONO. Given this possibility, we sought to determine whether G-quadruplex motifs are more abundant in NEAT1 sequence than would be expected by chance. We found the mean number of QGRS among a series of randomly generated sequences of the same length and GC content as NEAT1 to be 38.4 ± 1.99 (SEM), while human NEAT1 contains 111 (Figure 7A, B). The genomic arrangement (13,32,98) and paraspeckle-seeding function of mouse (15,24,26,28) and opossum (13) NEAT1 homologues is well conserved despite bearing little resemblance to the human NEAT1 sequence. With striking similarity to that seen with human NEAT1, our analysis found that mouse and opossum NEAT1 homologues contain 105 and 114 QGRS while only 53.2 ± 1.72 and 48.8 ± 2.25 would be expected by chance for sequences of equivalent length and GC content (Figure 7C,D). Despite very low sequence homology, enrichment of G-quadruplex motifs appears to be a feature common among NEAT1 homologues, as might be expected of a feature important for recruiting NONO and seeding paraspeckle assembly.

## DISCUSSION

Membraneless bodies play critical roles in homeostasis and have been implicated in a range of pathologies. While the phenomena guiding their formation and persistence are only beginning to be understood at a molecular level, evidence increasingly suggests that protein-RNA interactions play a central role. The manner by which specific proteins and RNA species coalesce within the crowded intracellular milieu to produce complex ordered structures is not well understood. With a well-defined structural arrangement initiated by a single RNA species, paraspeckles provide an ideal archetype for understanding the mechanistic basis and influence of these protein-RNA interactions. The biochemical basis of DBHS protein recruitment by NEAT1, the foundational step seeding paraspeckle formation, has been elusive. Low conservation of NEAT1 sequence has complicated identification of binding elements and hinted at the importance of secondary structure. This work provides the first evidence of a structural element which may fulfill these predictions.

Affinity of SPFQ and PSPC1 for G-rich RNA has not been previously observed. Enrichment of these proteins by G-rich and G-quadruplex RNA in the present study may have resulted from indirect enrichment through previously described heterodimerization with NONO (86,99). Future application of the *in vitro* assays used here to homogenous SFPQ and PSPC1 preparations may help to distinguish these binding activities. The finding that NONO recognizes G-rich RNA with preference for G-quadruplex structures is consistent with and adds a layer of structural specificity to known NONO RNA binding activities. Previous efforts to understand how NONO is targeted to RNA have uncovered binding preferences for two categories of RNA sequence. NONO was found to bind the purine-rich (R-rich) sequences of 5′ splice site RNA (80,94,100) and U5 snRNA (101). Additionally, binding assays (80,81) and systematic evolution of ligands by exponential enrichment (SELEX) (82) have identified an affinity of NONO for G-rich RNA sequences. Consistent with an affinity for G-rich RNA, several proteomic screens have found NONO among proteins associated with GGGGCC repeat RNA (62,77,102). Notably, a mitotic phosphorylation event severely limits the ability of NONO to bind to R-rich RNA but has no effect on binding to G-rich RNA (80). Reports that NONO has two distinct binding activities raise the possibility that each of its two RRMs is involved in a separate binding activity (80,84). RRM1, with a canonical RRM fold and RNA interaction motif (84), is sufficient for binding the R-rich 5′-splice site RNA (80,94). RRM2 lacks three of four aromatic residues normally required for RRM-mediated RNA binding and contains atypical but highly-conserved loop sequences longer than those found in most RRMs; if RRM2 does bind RNA, it does so in a previously uncharacterized manner (86). A proposed G-quadruplex-interaction motif recently defined through computational work (103) maps to RRM2, suggesting a potential role for RRM2 in RNA binding. Our finding that NONO does, in fact, bind G-quadruplexes with structural specificity lays the groundwork for further exploration of these potentially bipartite RNA binding activities. We hypothesize that G-quadruplex-binding activity is a previously unrecognized layer of structural specificity displayed by the functional unit responsible for previously reported G-rich binding activity. Future efforts to test the role of RRM2 in this activity will require careful consideration of the domain structure and its neighboring environment, as RRM2 contacts with other core DBHS domains are likely critical for the stability of obligate DBHS dimers (86).

Paraspeckle formation involves a complex series of events guided largely by interaction of paraspeckle proteins with NEAT1 (24,25,31). A critical early step in paraspeckle assembly is the recruitment of DBHS proteins to NEAT1, seeding formation of primary NEAT1 RNPs which are subsequently assembled to produce mature paraspeckles (Figure 7E) (24–28). Due to a lack of NEAT1 sequence conservation (32,37), it has been predicted that structural elements, as opposed to well-defined primary sequences, are responsible for NEAT1 recruitment of proteins in these initial stages of paraspeckle seeding (34,36,38). In the present study, the finding that NONO recognizes G-quadruplex structures spurred the hypothesis that G-quadruplex elements on NEAT1 are involved in NONO recruitment. We find an abundance of G-quadruplex motifs along the length of NEAT1, and that NONO shows preference for the G-quadruplex structure in binding to G-rich NEAT1 segments. Furthermore, we report evidence that G-quadruplex motifs mediate NONO-NEAT1 association in the findings that NONO binds to NEAT1 *in vivo* largely through G-quadruplex-forming sequences and that native NONO-NEAT1 association is disrupted by a G-quadruplex-distorting small molecule. Surprisingly we find that enrichment in G-quadruplex motifs is a feature conserved among NEAT1 homologues, in contrast to the low sequence conservation which has so far precluded identification of functional motifs. NONO recognition of NEAT1 through G-rich segments, as is suggested by these findings, is consistent with previous work showing that a mitotic phosphorylation event which inhibits R-rich binding activity but does not affect interactions with G-rich RNA similarly fails to affect NONO-NEAT1 association (80). G-quadruplex motifs, which our evidence suggests are conserved features of NEAT1 that mediate interactions with NONO, provide a promising candidate to fulfill the prediction that structural elements are responsible for protein recruitment to NEAT1 in the initial steps of paraspeckle formation (Figure 7E). The highlighting of these structures as possible functional elements provides a basis for future studies to assess their contribution to paraspeckle formation *in vivo*.

The model that NEAT1 G-quadruplexes recruit NONO in seeding paraspeckle assembly is supported by the recent finding (102) that GGGGCC repeat RNA can act as a functional substitute for NEAT1, inducing formation of paraspeckle-like bodies independent of NEAT1. This seeding activity of GGGGCC repeat RNA provides an opportunity to better understand the properties which imbue an RNA species with this activity. As we and others have previously reported that GGGGCC RNA forms stable G-quadruplexes (62–64), the present finding that NEAT1 contains abundant G-quadruplex motifs provides a feature common to both RNAs. The findings that NONO recognizes G-quadruplexes formed by both GGGGCC repeats and NEAT1 and that this activity mediates NONO-NEAT1 association suggest that G-quadruplexes contribute to seeding activity through mediating a critical step in paraspeckle seeding. Liquid-liquid phase separation is an important aspect of paraspeckle formation (20,25,27). Further supporting a role of G-quadruplexes in paraspeckle formation, GGGGCC repeat RNA promotes phase separation of RNA binding proteins including NONO and other paraspeckle components, and this activity is enhanced by G-quadruplex formation and inhibited by addition of TMPyP4 (104).

Taken together, the present findings reveal a previously unrecognized layer of structural specificity in the RNA-binding activity of NONO, with possible implications for an array of biological functions assigned to the protein. These functions include a central role in paraspeckle formation, where co-transcriptional recruitment to NEAT1 is a critical step in initiating paraspeckle assembly. The elements of NEAT1 responsible for this recruitment have long been a topic of interest, but their identification has been hindered by the low sequence conservation of NEAT1. In this study, we uncover evolutionarily conserved enrichment of G-quadruplex motifs on NEAT1 and present evidence that the structure-specific recognition of these motifs by NONO is important to NONO-NEAT1 association. Based on these findings, we present G-quadruplex structures as a promising candidate for the elusive recruiting elements of NEAT1 and lay the groundwork for future studies to interrogate the role of these structures in paraspeckle formation and function.

## Supplementary Material

gkaa475_Supplemental_Files

## Notes

Present address: Eric A.J. Simko, Messenger RNA Regulation and Decay Section, RNA Biology Laboratory, Center for Cancer Research, National Cancer Institute, Frederick, MD, USA.

Present address: Aaron R. Haeusler, Department of Neuroscience, Jefferson Weinberg ALS Center, Vickie and Jack Farber Institute for Neuroscience, Jefferson University, Philadelphia, PA 19107, USA.
