# Supplementary material for: G-quadruplexes offer a conserved structural motif for NONO recruitment to NEAT1 architectural lncRNA
Source: Nucleic Acids Res. 2020 Jun 4;48(13):7421–38. doi: 10.1093/nar/gkaa475 (PMC7367201; doi:10.1093/nar/gkaa475)
Supplement: gkaa475_Supplemental_Files [file gkaa475_supplemental_files.zip › Supplementary Materials.pdf]

Supplementary Data

G-quadruplexes offer a conserved structural motif for NONO recruitment to NEAT1 architectural lncRNA

Eric A.J. Simko, Honghe Liu, Tao Zhang, Adan Velasquez, Shraddha Teli, Aaron R. Haeusler, Jiou Wang

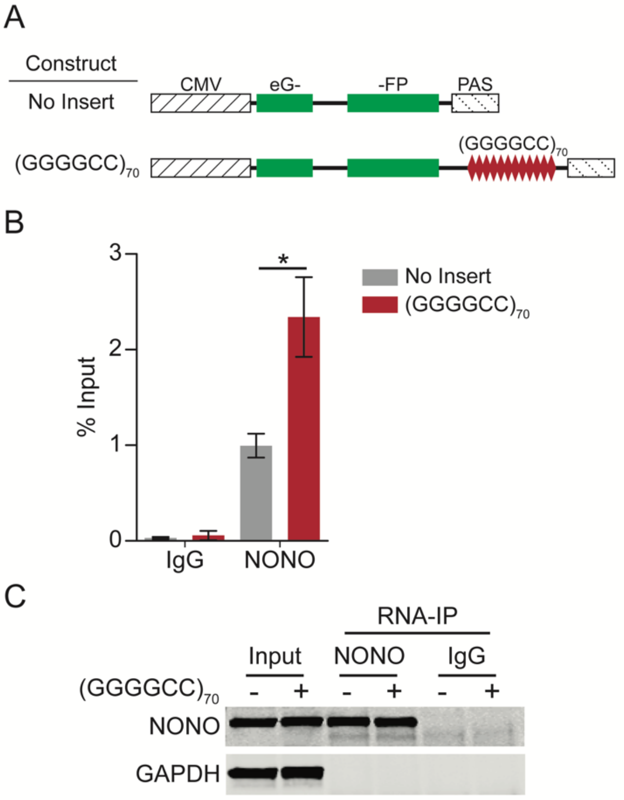

Figure S1. GGGGCC repeats recruit NONO in an alternative model transcript system. (**A**) An alternative eGFP-encoding model transcript system was used to test whether the presence of GGGGCC repeat sequence affects NONO-transcript association in a cellular context. The control transcript (“No Insert”), containing an eGFP open reading frame in two exonic segments with a β-globin-derived intron, is transcribed under the control of a cytomegalovirus (CMV) promoter and bovine growth hormone polyadenylation signal (PAS). In the $(GGGGCC)_{70}$ construct a sequence of 70 GGGGCC repeats was inserted in the 3’-untranslated region. (**B**) RNA-IP for NONO was performed on HEK293T cells transfected with the model transcript constructs, followed by RT-qPCR to compare enrichment of the model transcripts. Transcript enrichment is expressed as percentage of input with SEM (n = 4). “*” indicates $P < 0.05$ (two-tailed t-test). (**C**) Western blot of NONO IP fractions, 5% input, and enriched fraction from IP with IgG control. GAPDH is blotted as a negative control for IP enrichment.

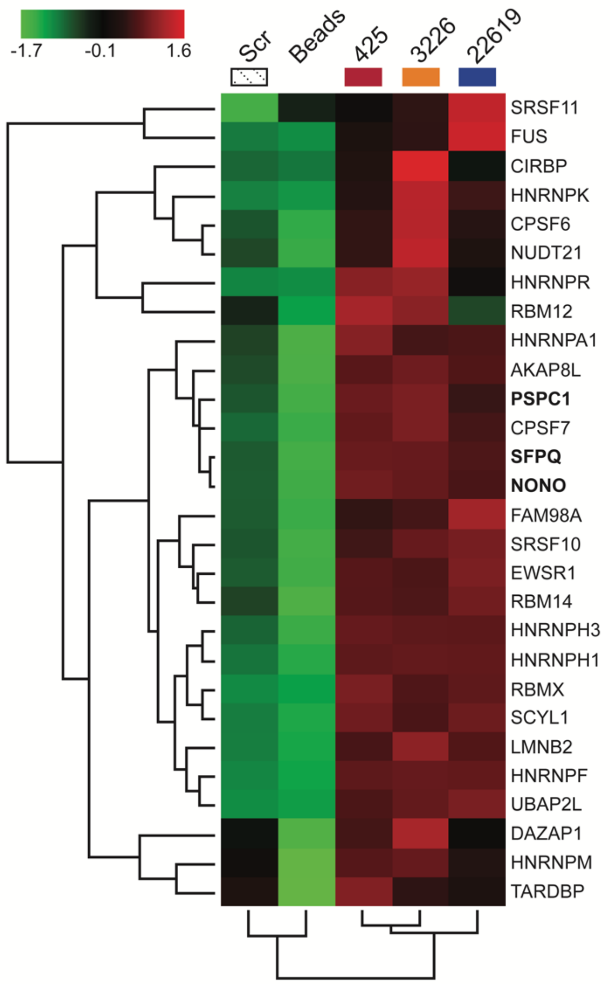

Figure S2. Paraspeckle proteins enriched by pulldown with NEAT1 G-quadruplexes. Heat map of paraspeckle protein enrichment by pulldown with NEAT1 RNA G-quadruplexes and G-rich non-G-quadruplex-forming RNA ('Scr'). Proteins and treatments were grouped by Euclidean clustering with complete linkage based on protein abundance, with values scaled before clustering. (See Supplementary Table S1 for complete list of identified proteins)

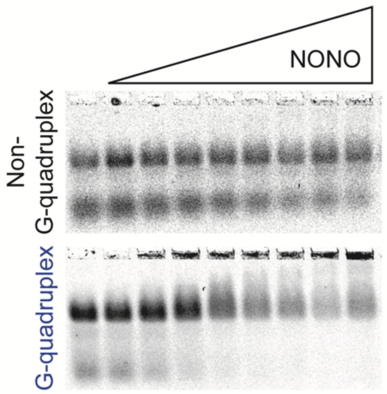

Figure S3. Full-length NONO preferentially interacts with NEAT1_22619 RNA in the G-quadruplex conformation.
EMSA with recombinant full-length NONO and NEAT1_22619 RNA in G-quadruplex and non-G-quadruplex conformations. 10 nM labelled NEAT1_22619 RNA was incubated with full-length NONO at the following concentrations (left to right): 0, 31.3 nM, 62.5 nM, 125 nM, 250 nM, 375 nM, 500 nM, 750 nM, 1.0 uM.

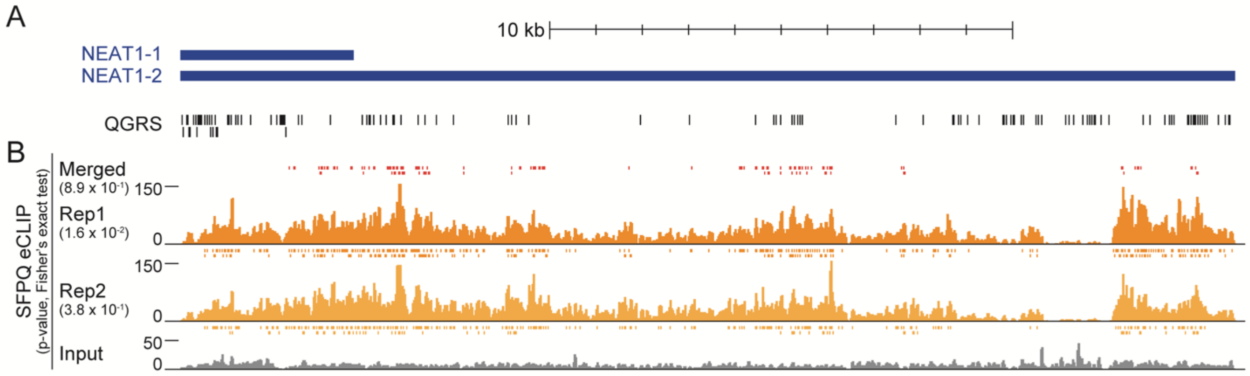

Figure S4. SFPQ binding sites along NEAT1.
(**A**) Quadruplex-forming G-rich sequences (QGRS) mapped to NEAT1 are shown as tall bars (black). (**B**) SFPQ eCLIP data mapped to NEAT1. Reads are plotted by intensity and called peaks are shown as short bars. Tracks, from top to bottom: Merged peaks, determined by replication and adjustment for input (dark orange); Replicate 1 read intensity plot with called peaks directly beneath (orange); Replicate 2 read intensity plot with called peaks directly beneath (light orange); size-matched paired input read intensity plot (grey). The statistical significance of overlap between QGRS and each set of peaks was determined using the two-tailed Fisher's exact test, with p-values shown in parentheses beneath each corresponding track label.

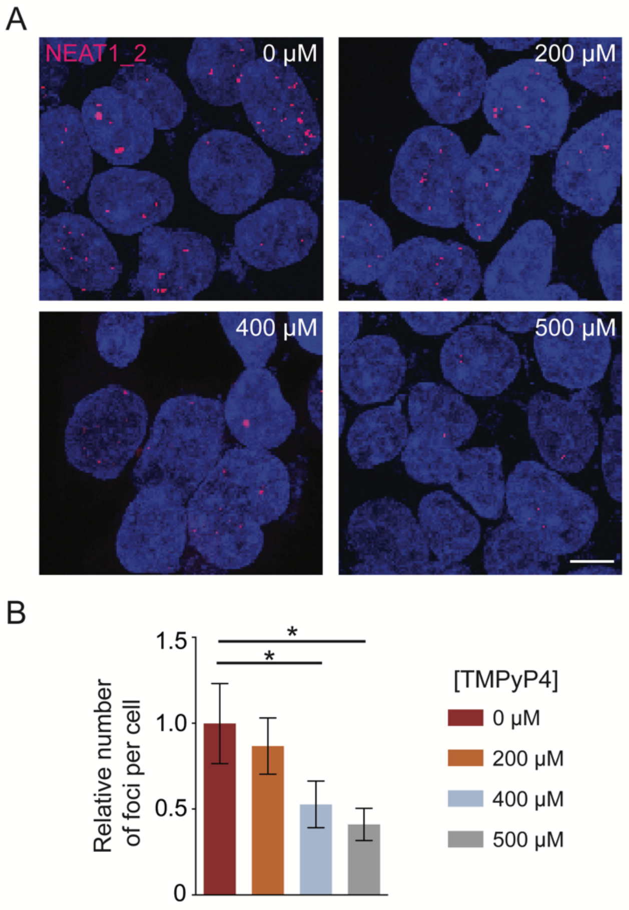

Figure S5. Treatment with TMPyP4 decreases the number of NEAT1 foci per cell.
(**A**) RNA FISH was used to detect NEAT1 as a marker for paraspeckles in HEK293T cells treated with increasing concentrations of TMPyP4. DAPI staining is shown in blue and NEAT1 staining is shown in red. Representative images for each treatment are shown. Scale bar: 5 μm. (**B**) Fold-change in NEAT1 foci/cell relative to control is plotted for each treatment. Mean values from three independent experiments are plotted with SEM. "*" indicates $P < 0.05$ (one-tailed t-test).

Supplementary Table S1. Proteins identified by mass spectrometry analysis as interactors of NEAT1 RNA G-quadruplexes. (Excel Spreadsheet)
Using mass spectrometry with quantitative comparisons through tandem mass tag (TMT) labelling, proteins that were pulled down with NEAT1 G-quadruplex-forming RNAs, G-rich non-G-quadruplex-forming RNA ('Scr'), and the bead-only control were identified and analyzed.
